# Latent Insanity

**Published:** 1880-05

**Authors:** 


### LATENT INSANITY

RESULTING from a morbid condition of the nervous system, superinduced by confinement to close rooms, by eating too much, by cherishing secret passions, appetites, propensities, whether of revenge, or hatred, or envy, by dwelling on imaginary slights, inattentions, indifferences and the like, is not a very uncommon calamity in general society. It is a very terrible social and domestic evil, making a clean wreck, as it sometimes does, of whole families.

It is a form of Insanity which comes on by the most imperceptible degrees. The very existence of it is now and then hinted at by a half playful, half chiding expression, "Why! you must be crazy." Months pass on, and sometimes years, but they bring with them their sure accretions. The symptoms become intensified, and decidedly foolish things are done, and affection exclaims, "O! he is only trying to be singular." "She affects eccentricity." In a long stretch of years, there is a link with the mad house here.

Sometimes, this malady presents a strong contrast in the character of the individual. He may perform most of the duties of social and commercial life with the utmost propriety, and with scrupulous exactness, while at the same time, he exhibits antipathies and cherishes dislikes and suspicions against the closest friends and the nearest relations of life, and yet for years, this mad mask may be so adroitly worn, that no tangible suspicion of active disease is entertained, until some terrible transaction reveals the unwelcome truth, in all its horror—HE'S MAD!

There is many an unrecognized maniac loose in society, who, in the language of high medical authority, acting under some one predominant morbid idea, may bring destruction into a home once beautiful and a household once happy. Such a man may become a tyrant, a brute, a spendthrift, a suicide, and yet pass through life as a rational and healthy man. What we charitably call "*Eccentricity*" is but too often, hidden madness. Such persons are curious in their ideas of dress, walk singularly and use a great variety of odd expressions; and yet, in other respects, they exhibit such an acuteness of intellect, such a lightning-like rapidity of perception, as to the proprieties of things, we cannot bring ourselves to believe that there is insanity there; and we wait and hope on. But look a little more closely. Their temper is furious, utterly uncontrollable. The slightest occurrence makes all the difference between a lamb and a lion, so of the man; while in the woman, naturally and in health, all that a woman should be, kind, loving, gentle, humane, pitying and pure, the most trifling causes wake up an ungovernable fury, and every thing like feeling, and sentiment and refinement, in speech and action, disappears in an instant, and there stands before us, an angel ruined!

By all our love for human happiness therefore, we say to every reader, especially to those who have authority over others, discourage everything like a love of seclusion in your children. Do not leave young people much alone. We doubt much the propriety of allowing any member of a family "a room to themselves." The children of a household should be taught never to lock their doors on the inside, for more reasons than one. The feeling should abide on us all, day and night, "YOU ARE OBSERVED." O, what a vast amount of wrong doing and crime, the prevalence of that single idea would prevent! Let young girls and boys be made to learn, that every box and drawer and letter and book and port folio, must be left accessible, always and under all circumstances, to a loving mother's eye.

There is a wise lesson here, of infinite value, for it will save many a mother's brain from wreck, if properly heeded. Especially do we address ourselves to young wives. Don't pout. Don't shut yourself up in a room and cry by the hour for some trifling inadvertency or imaginary neglect of your husband. He has won you and now has a living to win. He has to deal in the rough world, and jostle and be jostled by rough men. He has money to pay, and to keep up his credit and his honor, he has it to earn, or having earned it once, he may have to earn it a second time by endeavoring to collect it. In these things he has daily disappointments, and severer losses. He loves you, and in that love, seeks to confine trouble to his own bosom, and thus many a time his mind is away from himself, as well as you, and any neglect of you, is a necessity, not an intention. But feed the feeling! Take counsel of passion! and you fan a spark, which lights up a disease which, but too often, locks you up in an asylum.

